# Mobile Health Interventions for Improving Colorectal Cancer Screening Rates: A Systematic Review and Meta-Analysis

**DOI:** 10.31557/APJCP.2021.22.10.3093

**Published:** 2021-10

**Authors:** Anton Elepaño, Alyssa Samantha Fusingan, Eric Yasay, Jereel Aron Sahagun

**Affiliations:** 1 *Department of Medicine, University of the Philippines-Philippine General Hospital, Manila, Philippines. *; 2 *Division of Gastroenterology, Department of Medicine, University of the Philippines-Philippine General Hospital, Manila, Philippines. *

**Keywords:** Colorectal neoplasms, telemedicine, early detection of cancer

## Abstract

**Objective:**

The aim of this systematic review and meta-analysis was to determine the efficacy of different mHealth interventions in increasing colorectal cancer (CRC) screening rates.

**Methods:**

A literature search for eligible studies was done in ClinicalTrials.gov, PubMed, and Scopus in October 2020. Included studies were randomized controlled trials done on adults due for CRC screening, who received either an mHealth intervention to promote screening or usual care. The primary outcome from these studies was completion of CRC screening. Two reviewers independently worked on selecting studies, collecting data, and determining risk of bias. Adjusted odds ratios (AOR) for CRC screening rates were summarized into a Forest plot.

**Results:**

A total of ten trials from three continents were included in the qualitative analysis. Risk of bias is low in terms of randomization, but high in terms of participant blinding, due to the nature of the interventions. Meta-analysis of four trials showed low clinical and statistical heterogeneity (I^2^=0%). Overall, the use of mHealth interventions is associated with higher CRC screening uptake when compared to usual care (AOR 1.33; 95% CI, 1.20-1.46). This effect was seen across different types of mHealth interventions, which included automated and non-automated telephone education and text-message reminders.

**Conclusion:**

This study showed that mHealth is associated with increased CRC screening participation regardless of the type of intervention used.

## Introduction

Mobile health (mHealth) is loosely defined by the World Health Organization as “medical and public health practice supported by mobile devices, such as mobile phones, patient monitoring devices, personal digital assistants, and other wireless devices” (Kay et al., 2011). Applications of mHealth include healthy lifestyle promotion, diagnosis and education, healthcare facility finder, and appointment reminders (Silva et al., 2015). Compared with face-to-face delivery of health services, mHealth offers advantages in terms of accessibility, cost, and the option to send out automated reminders (Silva et al., 2015; Moset et al., 2010). Because of the ubiquity of mobile phones, its role for health screening programs has been a focus of interest.

Colorectal cancer (CRC), which is the second most common cause of cancer deaths worldwide, is among the cancers that may benefit most from screening methods (Sung et al., 2021). If detected in its earliest stage, more than 90% of patients with colorectal cancer survive beyond five years (Gomez et al., 2007). Current screening methods include laboratory-based tests such as fecal occult blood testing, or office-based procedures such as sigmoidoscopy or colonoscopy. Screening programs come in the form of population-based programs, in which individuals at increased risk for colorectal cancer are actively identified and contacted; or in the form of opportunistic programs, which rely on the initiative of the healthcare provider to encourage patients to participate in screening (Gomez et al., 2007). 

There have been prior systematic reviews which looked at the effects of specific mHealth interventions on colorectal screening rates (Uy et al., 2017; Bai et al., 2020; Tsipa et al., 2020). In a systematic review done in 2017, text-messaging was shown to have a small effect on colorectal screening rates (increase in absolute screening rates ranging from 0.6 to 3.3%) (Uy et al., 2017). Based on a pooled analysis of five studies done in 2018, tailored intervention delivered via telephone counselling and print material showed benefit in increasing colonoscopy screening rate (OR, 2.21; 95% CI, 1.71-2.85) (Bai et al., 2020). A meta-analysis conducted in 2016 also showed increased screening uptake with use of remote contact interventions (OR, 1.45; 95% CI, 1.38-1.53) (Tsipa et al., 2020). However, none of these studies paid particular focus on the different mHealth modalities available. Moreover, a number of randomized controlled trials on these topics have been completed since the conduct of these systematic reviews. Hence, this meta-analysis will build upon previous knowledge and help consolidate new evidence specifically on mHealth interventions for colorectal cancer screening. Therefore, the aim of this systematic review and meta-analysis was to compare the efficacy of mHealth interventions with local standard of care in increasing CRC screening rates among eligible adults.

## Materials and Methods

This systematic review was conducted and reported following recommendations from the PRISMA (preferred reporting items for systematic review and meta-analysis) statement (Liberati et al., 2009).

*Eligibility criteria*

Only randomized controlled trials (RCT) involving participants who are due for CRC screening, i.e., adults at least 50 years of age with average risk for CRC and those with above average risk for having a family history of colon cancer or polyps were included. Trials were considered if the experimental group received an mHealth intervention designed to promote CRC screening which may include electronic messages delivered through a mobile phone, telephone calls offering basic patient education, web-based counselling, or mobile apps with a decision support system. The comparator in these trials should be the standard of care in the institutions where they were conducted, and may include face-to-face CRC screening education, brochures, and other informational materials. Trials employing a screening promotion method other than mHealth, e.g., mailed out test kits with phone call follow-ups, were excluded for possible confounding.

The primary study outcome of interest was the completion of a CRC screening method such as colonoscopy, flexible sigmoidoscopy, fecal immunochemical test (FIT), or fecal occult blood test (FOBT) (based on medical records or chart documentation). Secondary outcomes were recorded as well.

*Electronic searches*

A literature search was performed on the following databases with no restriction on time or language of publication: PubMed, Scopus, and ClinicalTrials.gov. Search strategies employed a combination and variation of the following keywords: colorectal cancer, telehealth or mHealth, and cancer screening. The detailed search strategy is available as a supplementary file.

*Selection of studies*

Two reviewers worked independently in screening records identified in the electronic search. The predetermined inclusion criteria for eligible studies were applied to the titles and abstracts, and then to the full text reports. Discrepancies in selected studies between the two reviewers were discussed and resolved by consensus or by consultation with a third reviewer. The process of excluding studies was documented.

*Data collection*

Data collection was done independently by two reviewers. Discrepancies were discussed and resolved with the help of a third-person adjudicator as necessary. Standardized data extraction forms were used to report the study designs, participants, interventions, comparators, and primary and secondary outcomes for each of the selected studies.

*Risk of bias assessment*

Risk of bias was assessed independently by two reviewers with a third reviewer as adjudicator. The following domains were evaluated: (1) random sequence generation (selection bias), (2) allocation concealment (selection bias), (3) blinding of participants and personnel (performance bias), (4) blinding of outcome assessment (detection bias), (5) incomplete outcome data (attrition bias), and (6) selective reporting (reporting bias). A graphic representation of the risk of bias assessment was generated in Review Manager 5.4.

*Data synthesis and statistical analysis*

The preliminary search showed a number of eligible studies presenting outcomes as odds ratio with statistical adjustments for age, sex, geographic area, and receipt of previous CRC screening. Adjusted odds ratios (AOR) were pooled using the generic inverse variance method with a corresponding 95% confidence interval. The variables that were adjusted for in each study were recorded. Clinical heterogeneity was assessed by looking at variability across studies in terms of patient demographics, clinical circumstances, and comparability of interventions applied. Studies were pooled if no significant clinical heterogeneity was noted. Statistical heterogeneity was assessed by determining the chi-square test and I2 statistic. An I2 value of more than 50% was interpreted as substantial heterogeneity. All statistical analyses were done using Review Manager 5.4.

## Results

**Figure 1:**
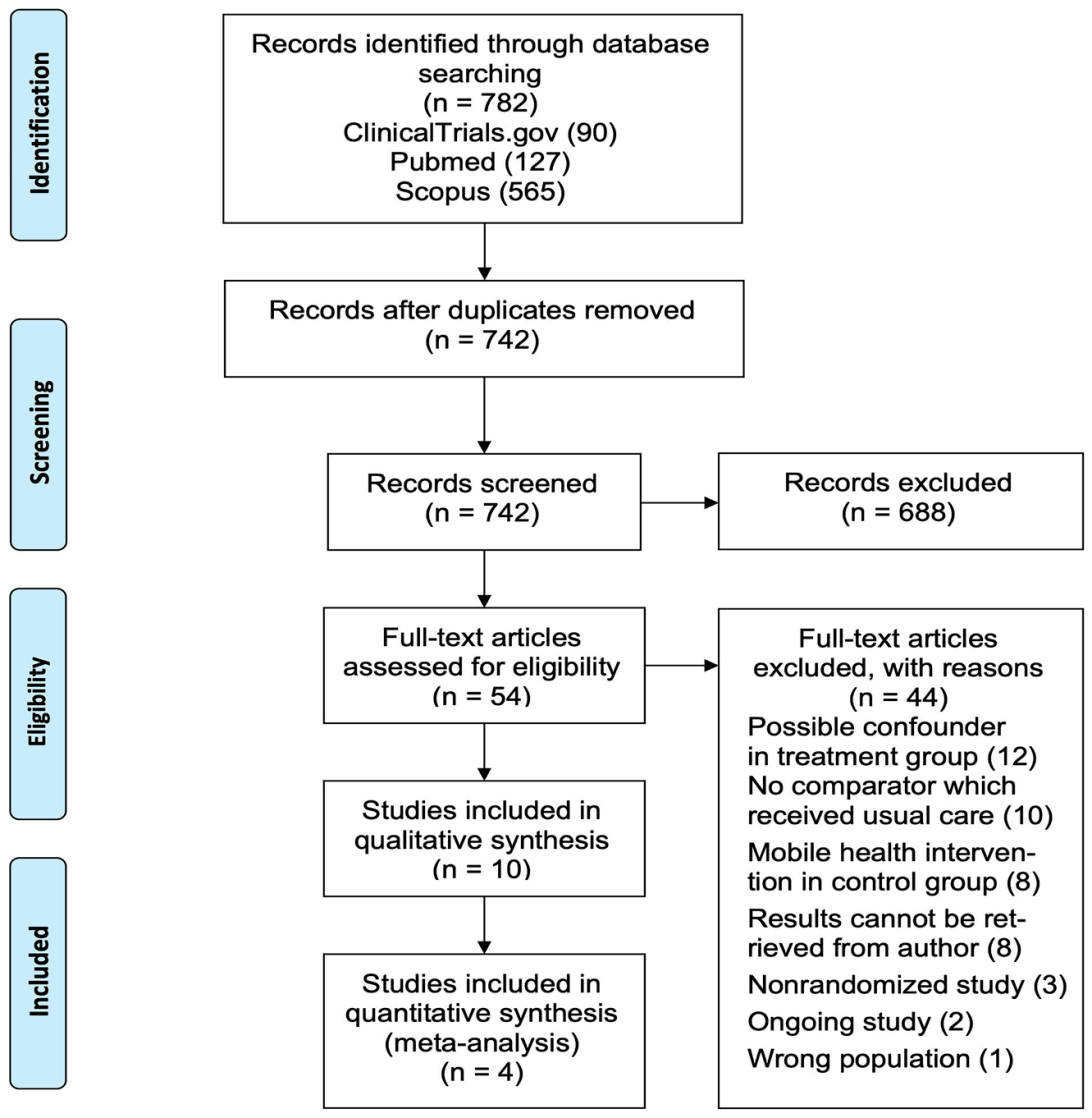
Study Flow Diagram

Literature search from three databases of published and unpublished trials was conducted in October 2020. The study flow diagram is presented in Figure 1. The initial search yielded 782 records, from which 40 duplicates were removed. Screening of titles and abstracts resulted in the removal of 688 records which were deemed to be not relevant. Full texts of the remaining 54 trials were retrieved and appraised for eligibility based on predefined criteria. Ten trials were included in the qualitative analysis and four trials were included in the final meta-analysis after assessing clinical heterogeneity.

Characteristics of each study are outlined in Table 1. All of the studies included were randomized controlled trials and were published in the English language. Five studies came from North America, four from Europe, and one from Asia. All of the included studies enrolled patients who were due for CRC screening. None of the studies enrolled first-degree relatives of people with CRC. The mHealth interventions were different among trials. Telephone education was used in eight of the trials, text-message reminder was used in two trials, while one trial employed web-based education. Usual care was the comparator used in all studies; however, local practices varied among study sites. The primary outcome for all studies was receipt of CRC screening. Screening types included stool-based tests such as FOBT and FIT, and office-based tests such as colonoscopy. CRC screening rates were consistently higher with mHealth intervention compared to usual care except for one study which used face-to-face consult as the comparator (Stoop et al., 2012).

**Figure 2:**
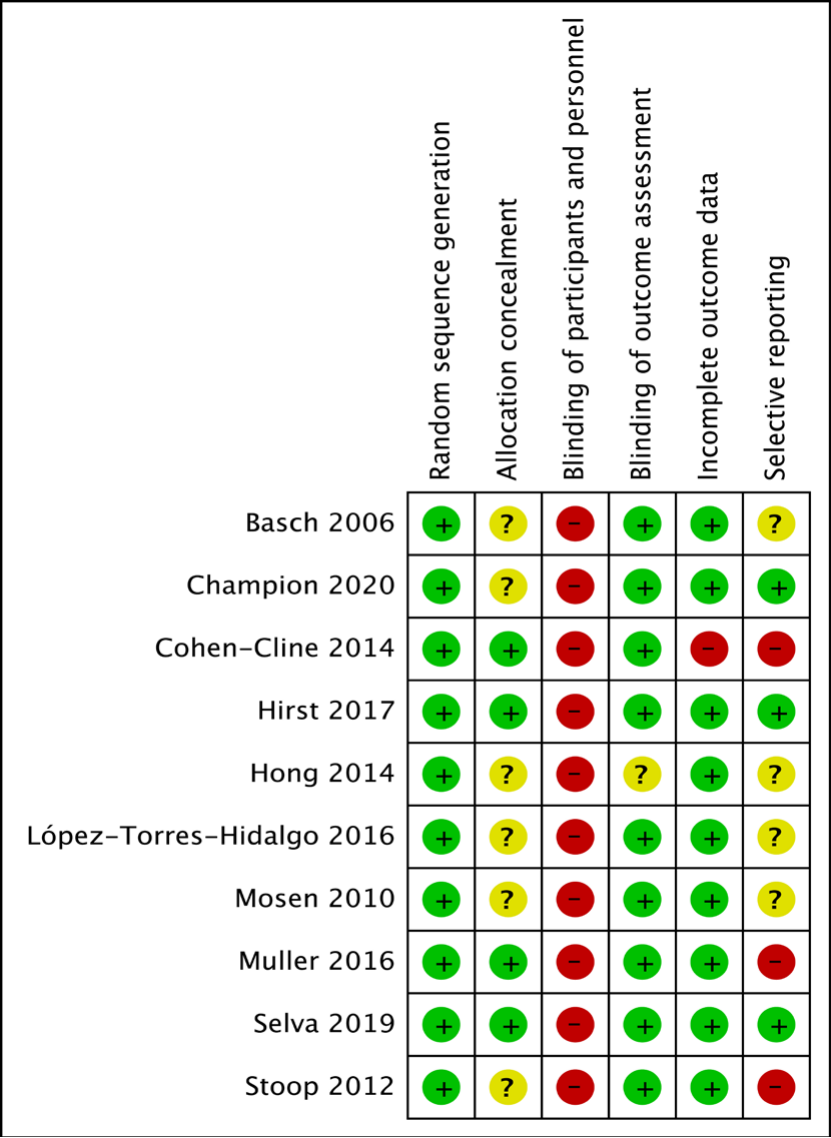
Risk of Bias Summary Using Review Manager 5.4

**Figure 3:**
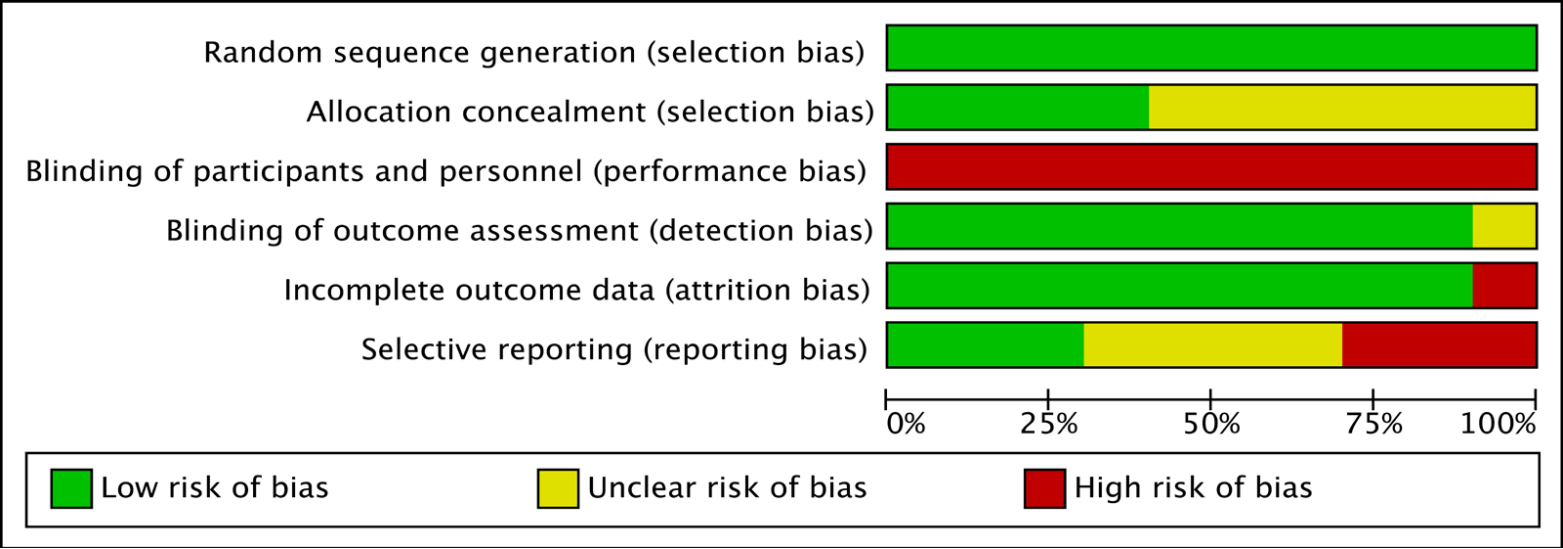
Risk of Bias Assessment Presented as Percentages Across the Ten Included Studies

All ten studies were assessed to be of low risk of bias in terms of random sequence generation, yet of high risk of bias in terms of blinding participants and personnel (Figure 2). Ninety percent of studies were assessed to be at low risk for detection and attrition bias. Allocation concealment and selective reporting were unclear for the majority of the included studies (Figure 3).

Among the ten studies reviewed, six reported adjusted estimates to control for baseline variability in population characteristics and to account for potential confounding covariates. These studies are presented in Table 2. Variability among the four trials which were not included in the quantitative analysis was noted to be significantly high (I^2^=97%).

**Figure 4.**
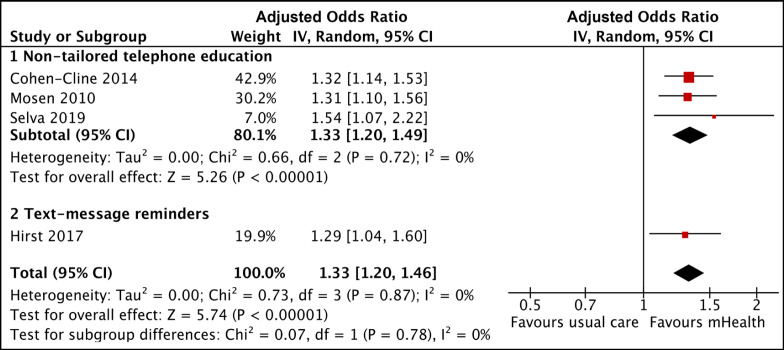
Pooled Analysis of AOR for CRC Screening Rate

Two trials, deemed to have significant clinical variability for having exclusively enrolled only either sexes, were not included in the final meta-analysis (Champion et al., 2020; Hong and Kam, 2014). The effect estimates of the remaining four studies are summarized in Figure 4. In the first three studies presented (Cohen-Cline et al., 2014; Mosen et al., 2010; Selva et al., 2019), non-tailored telephone education showed a pooled AOR of 1.33 with 95% confidence interval (CI) of 1.20-1.49. Overall, accounting for a fourth trial by Hirst et al. (2017) which used text-message reminders, the use of mHealth interventions is associated with higher CRC screening uptake when compared to usual care (AOR, 1.33; 95% CI, 1.20-1.46). The trials included in the meta-analysis employed a mix of automated interactive voice calls (Cohen-Cline et al., 2014), text-message reminders (Hirst et al., 2017), telephone reminders (Mosen et al., 2010), and brief informative phone calls (Tsipa et al., 2020). Despite these differences and after adjusting for possible confounding factors, the effect size of the interventions remained comparable with a low statistical heterogeneity across the four studies (Chi^2^ 0.72, P=0.87; I^2^=0%).

**Table 1:** Characteristics of Included Randomized Controlled Trials

Study	Population	Exposure	Control	Outcome	No. screened/no. of participants	
					Usual care	mHealth
Tailored telephone education						
1. Basch, et al. (2006)	52- to 79-year-old males and females in New York, US due for CRC screening	Tailored telephone education Median frequency of calls: 5 Median duration of calls: 23.5 min	Usual care: printed educational materials for CRC screening	Receipt of FOBT, colonoscopy, or proctosigmoidoscopy within 6 months	14/230 (6.1%)	61/226 (27.0%)
2. Champion, et al. (2020)	51- to 75-year-old females in Louisiana, US due for CRC and breast cancer screening	Tailored telephone education Average duration of calls: 19 min	Usual care: may receive printed reminders for cancer screening	Receipt of CRC screening within 6 months	23/131 (17.6%)	153/376 (40.1%)
Non-tailored telephone education						
3. Cohen-Cline, et al. (2014)	50- to 81-year-old males and females in Washington, US due for CRC screening	Automated non-tailored telephone education Average duration of calls: 5 min	Usual care: annual printed reminder for cancer screening	Receipt of FOBT or colonoscopy at 6 months	234/3005 (7.8%)	803/10000 (8.0%)
4. Hong and Kam (2014)	50- to 59-year-old males in Daegu, South Korea due for stomach cancer and CRC screening	Non-tailored telephone education Duration of calls: not reported	Usual care: printed educational materials for CRC screening	Receipt of CRC screening within 3 months	30/240 (12.5%)	59/243 (24.3%)
5. López-Torres-Hidalgo, et al. (2016)	50- to 74-year-old males and females in Albacete, Spain	Non-tailored telephone education Duration of calls: not reported	Usual care: received no information	Receipt of CRC screening within 2 years	25/423 (5.90%)	122/423 (28.80%)
Non-tailored telephone education						
6. Mosen, et al. (2010)	51- to 80-year-old males and females in Denver, US due for CRC screening	Automated non-tailored telephone education with reminder Average duration of calls: 1 min	Usual care: dependent on healthcare provider; received no further information	Completion of FOBT screening within 6 months	521/3000 (17.4%)	703/3000 (23.4%)
7. Selva, et al. (2019)	50- to 69-year-old males and females in Catalonia, Spain for CRC screening	Non-tailored telephone education Duration of calls: 5.25 min	Usual care: mailed FOBT kit with printed reminders	Completion of FIT screening within 6 months	102/256 (39.8%)	122/256 (47.7%)
8. Stoop, et al. (2012)	50- to 75-year-old males and females in Amsterdam and Rotterdam, Netherlands	Non-tailored telephone education Duration of calls: 30 min	Usual care: face-to-face consultation	Receipt of colonoscopy (assessment duration not specified)	752/3298 (22.8%)	674/3302 (20.4%)
Text-message reminders						
9. Hirst, et al. 201720	60- to 74-year-old males and females in London, UK	Automated text-message reminder Frequency of text-message reminder: 1	Usual care: mailed FOBT kit with printed reminders	Completion of FOBT screening within 4 months (18 weeks)	1648/4135 (39.9%)	1674/4134 (40.5%)
10. Muller 201621	40- to 75-year-old males and females of Alaskan Natives and American Indian heritage in Anchorage, Alaska due for CRC screening	Automated text-message reminder Frequency of text-message reminder: 3	Usual care: dependent on healthcare provider	Receipt of FOBT or colonoscopy at 6 months	142/1193 (11.9%)	181/1193 (15.2%)

**Table 2:** Adjusted Odds Ratios and Variables Considered from Different Studies

Study	Adjusted odds ratio (95% CI)	Variables adjusted for
Champion, et al. (2020)	Stool-based screening:1.52 (0.85-2.73) Colonoscopy: 4.59 (2.24-9.42)	Mammography medical record indicator; health site; age; race; education; income; marital status; BMI; whether depression limits patient’s activities; family history of 1 or more blood relatives with colon cancer ; family history of 1 or more blood relatives with breast cancer; perceived risk of breast cancer; doctor’s recommendation for mammography; number of past-year primary care visits, excluding eye care and dentistry; number of self-reported health problems; baseline stage of readiness; and scale scores measuring knowledge, susceptibility, benefits, fear, fatalism, self-efficacy, and barriers
Cohen-Cline, et al. (2014)	1.32 (1.14-1.52)	Age, sex, and prior CRC screening
Hirst, et al. (2017)	1.29 (1.04-1.58)	Age, sex, Index of Multiple Deprivation, and Clinical Commissioning Group
Hong, et al., (2014)	2.07 (1.28-3.36)	Age
Mosen, et al., (2010)	1.31 (1.10-1.56)	Age, sex, and prior CRC screening
Selva, et al., (2019)	1.54 (1.07-2.20)	Age, sex, and geographic area

CI, confidence interval; CRC, colorectal cancer screening; BMI, body mass index

## Discussion

This systematic review and meta-analysis showed that the use of mHealth, in addition to usual care, is associated with an increase in CRC screening uptake by as much as 20-46% regardless of the type of intervention used. Potential sources of bias have been addressed in most of the trials reviewed. Electronic health records have been used to select patient populations and also automatically collect information on CRC screening rates without influence of outcome assessors (Hirst et al., 2017). However, due to the nature of mHealth interventions, blinding of participants was not feasible in all of the trials. 

Several factors have been reported to affect compliance with CRC screening, hence the majority of the studies adjusted for these variables. Participation rates for CRC screening have previously been reported to be lower among males, younger age groups, and lower educational level (Selva et al., 2019; Deding et al., 2017). This may account for the disproportionately higher uptake in an all-female population enrolled in one of the studies (Champion et al., 2020). In this systematic review, a post-hoc analysis of studies which used unadjusted models showed high heterogeneity (I^2^=97%) suggesting that population demographics may indeed be contributing to the variability in between studies. 

The findings in this study are consistent with previous systematic reviews in that remote contact methods (Tsipa et al., 2020), including text-message reminders (Uy et al., 2017) and tailored telephone interventions (Bai et al., 2020) among CRC susceptible individuals, are associated with an increase in CRC screening rates. Notably, in the meta-analysis by Tsipa et al. (2020), pairwise comparisons between face-to-face and mHealth interventions showed no significant difference from each other in terms of improving CRC screening rate (Tsipa et al., 2020). In times of limited face-to-face consults such as the current pandemic situation, mHealth may be an option to aid in improving CRC screening rates.

In contrast to the previous systematic reviews, the current study restricted the control group to usual care only, whereas the two previous reviews included studies that employed usual care, another intervention, or no intervention as a comparison group (Bai et al., 2020; Tsipa et al., 2020). The current study was also able to include more recent trials until 2020, as well as additional intervention types such as tailored and non-tailored telephone education. 

Although this meta-analysis has demonstrated the effectiveness of mHealth interventions in promoting CRC screening, there are certain limitations that must be considered. Firstly, in the recruitment process of the studies included, participants who agreed to join the trials were already more predisposed to participating in CRC screening, rather than those who opted out of study participation in the first place (Basch et al., 2006). Secondly, the desired effect of mHealth will have to be weighed against the expenses used to set up and maintain these interventions. In particular, its integration in countries without established CRC screening programs, may prove to be challenging. 

Included studies covered receipt of colorectal screening as its outcome, which included either mailed submission of stool FIT or FOBT or receipt of colonoscopy. Recommendations for further studies may explore the percentage of patients with positive FIT or FOBT who actually proceed with colonoscopy, as this is a more direct way of screening and preventing CRC.

In conclusion, mHealth interventions, such as telephone intervention and text messages, are associated with as much as 20-46% increase in colorectal cancer screening rates compared to usual care.

## Author Contribution Statement

AE and ASF were involved in the study design, literature search, selection of studies, data collection, risk of bias assessment, statistical analyses, interpretation of results, review of drafts, and approval of the final paper. EY and JAS contributed to the study protocol, statistical analyses, interpretation of results, review of drafts, and approval of the final paper.
